# High‐Precision Wavelength Tuning of GeSn Nanobeam Lasers via Dynamically Controlled Strain Engineering

**DOI:** 10.1002/advs.202207611

**Published:** 2023-04-18

**Authors:** Youngmin Kim, Hyo‐Jun Joo, Melvina Chen, Bongkwon Son, Daniel Burt, Xuncheng Shi, Lin Zhang, Zoran Ikonic, Chuan Seng Tan, Donguk Nam

**Affiliations:** ^1^ School of Electrical and Electronic Engineering Nanyang Technological University 50 Nanyang Avenue Singapore 639798 Singapore; ^2^ School of Electronic and Electrical Engineering University of Leeds Leeds LS2 9JT UK

**Keywords:** GeSn, lasers, tuning, identical lasers, silicon photonics, short‐ and mid‐wave infrared optoelectronics

## Abstract

The technology to develop a large number of identical coherent light sources on an integrated photonics platform holds the key to the realization of scalable optical and quantum photonic circuits. Herein, a scalable technique is presented to produce identical on‐chip lasers by dynamically controlled strain engineering. By using localized laser annealing that can control the strain in the laser gain medium, the emission wavelengths of several GeSn one‐dimensional photonic crystal nanobeam lasers are precisely matched whose initial emission wavelengths are significantly varied. The method changes the GeSn crystal structure in a region far away from the gain medium by inducing Sn segregation in a dynamically controllable manner, enabling the emission wavelength tuning of more than 10 nm without degrading the laser emission properties such as intensity and linewidth. The authors believe that the work presents a new possibility to scale up the number of identical light sources for the realization of large‐scale photonic‐integrated circuits.

## Graphical abstract

The technology to develop many identical coherent light sources on an integrated photonics platform holds the key to the realization of PICs. Herein, a scalable technique to produce identical on‐chip lasers by dynamically controlled strain engineering is demonstrated. By using localized laser annealing that can control the strain, emission wavelengths of several GeSn 1D photonic crystal nanobeam lasers are matched.

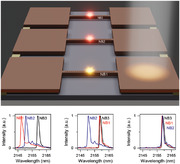

## 1 Introduction

Photonics‐based platforms have recently become the strongest candidate for realizing unprecedentedly powerful computing devices.^[^ ^1^, ^2^ ^]^ Particularly, integrated photonics allows putting together many photonic devices on a tiny chip,^[^ ^2^ ^]^ which is crucial to increasing the computational capacity of photonics‐based processors. Photons are crucial resources for such computation and they have to be identical in all degrees of freedom including the wavelength for achieving on‐chip interference,^[^ ^3^ ^]^ which is essential in performing computation. All of the recent demonstrations of integrated photonics‐based on‐chip computation used a bulky off‐chip laser^[^ ^4^, ^5^, ^6^ ^]^ that provides coherent photons into multiple waveguides on a chip because of the lack of the technology to build multiple identical lasers. Despite its significance, the development of identical light sources on photonic‐integrated circuits (PICs) has been hampered by subtle variations of material properties across a chip^[^ ^7^ ^]^ and the fabrication imperfection of optical cavities.^[^ ^8^ ^]^ A precise wavelength tuning capability is therefore deemed crucial to overcoming the spectral inhomogeneity of on‐chip light sources.

Over the past few years, several research groups reported wavelength‐tunable III–V light sources by modifying the material's physical properties^[^ ^7^ ^]^ or by controlling optical cavities.^[^ ^9^ ^]^ Despite much progress in wavelength tuning techniques in III–V material systems, however, their inherent incompatibility with the dominant complementary metal‐oxide‐semiconductor (CMOS) process limits the III–V laser technology from being widely adopted for PICs. Additionally, the previous work demonstrating the spectral alignment of quantum dot emitters^[^ ^7^ ^]^ requires the light‐emitting medium to be directly excited by high‐power lasers, which may potentially degrade the light emission properties. Furthermore, such tuning techniques have not been applied to on‐chip lasers, which are crucial for realizing PICs. Recently, GeSn alloys have been considered one of the most promising materials for realizing integrated lasers on PICs.^[^ ^10^, ^11^, ^12^, ^13^, ^14^, ^15^, ^16^, ^17^, ^18^ ^]^ Strain engineering has shown the potential to tune the emission wavelengths of GeSn lasers while tensile strain can also improve the laser characteristics by improving the directness of the energy band structure of GeSn.^[^ ^13^, ^14^ ^]^ However, the amounts of strain in each device in these previous demonstrations are pre‐set, making it impossible to develop identical laser sources emitting photons at the same wavelength.

Here, we present a scalable technology that allows matching the emission wavelength of separate GeSn lasers that are integrated on a single Si chip. By harnessing controllable and localized optical annealing that modifies the crystal structure of GeSn dynamically, our technique can precisely tune the mechanical strain in the laser gain medium and adjust the emission wavelength over a large spectral range of >10 nm. By applying this approach to several one‐dimensional (1D) photonic crystal nanobeam GeSn lasers whose initial emission wavelengths are considerably different, we experimentally demonstrate the ability to match the emission wavelengths of different lasers with sub‐nanometer precision. We believe that our simple and scalable method of achieving identical lasers in a fully CMOS‐compatible material system paves the way towards a monolithic realization of large‐scale PICs.

## 2 Results

### 2.1 Design of Dynamically Controlled Wavelength Tuning in GeSn Lasers

**Figure 1:**
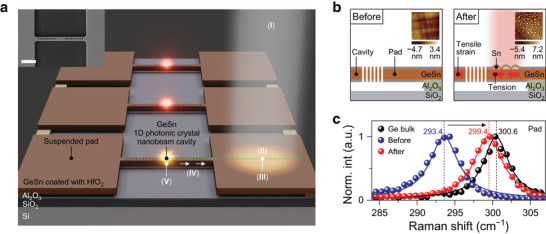
Design of dynamically controlled wavelength tuning in GeSn lasers. a) Schematic illustration showing how dynamically controlled wavelength tuning is achieved in a GeSn laser, which can be summarized in the five steps: (I) laser pumping at the pad for local annealing, (II) Sn segregation upon annealing, (III) tension generation due to Sn segregation, (IV) cavity pulled by the tensioned pad, and (V) tensile strain in the gain medium due to the pulling force in the pad. Inset: SEM image of the fabricated laser device. Scale bar, 3 µm. b) Cross‐sectional view of the schematic illustration along the green‐dashed line in Figure 1a before and after localized laser annealing. Sn‐rich clusters are formed at the surface of the pad after annealing. Inset: AFM topography of the pad area before and after annealing. The scan area is 2 × 2 µm^2^. Small dome‐shaped islands are observed on the annealed surface, whereas the unannealed surface is smooth. c) Raman spectra of the pad before (blue) and after (red) annealing. The peak positions of ≈293.4 and ≈299.4 cm^−1^ before and after the annealing indicate a reduction of Sn content in the GeSn pad after the annealing. A spectrum of Ge bulk (black) with a peak position at 300.6 cm^−1^ is shown for reference.

**Figure** **1**a presents a schematic illustration that explains how we achieve dynamically controlled wavelength tuning in our GeSn laser. The inset to Figure 1a shows a scanning electron microscope (SEM) image of the successfully fabricated laser device used in our study (see Note S1, Supporting Information, for more details on device design and fabrication process). Our device structure consists of a GeSn 1D photonic crystal nanobeam cavity and two suspended pads connected to the cavity. A 1D photonic crystal cavity allows confining light in the gain medium to achieve lasing.^[^ ^16^ ^]^ The suspended pads play a key role in enabling the emission wavelength tuning via a mechanism explained in the following steps: upon a localized laser annealing in the pad area by an external laser pumping (step I), the temperature of the annealed area rises beyond the critical temperature (*T*_c_) that induces the Sn segregation to the surface of the GeSn layer^[^ ^19^, ^20^, ^21^ ^]^ (step II). The Sn segregation is equivalent to a removal of large Sn atoms from the lattice of GeSn. The reduction of large Sn atoms generates tension in the lattice because the GeSn lattice is rearranged with fewer Sn atoms in the same volume (step III). The nanobeam cavity is pulled by the tensioned pad (step IV). As the nanobeam is pulled along both sides, the laser gain medium at the center of the nanobeam is tensile strained (step V) and the refractive index of the cavity is changed due to the strain‐induced bandgap shift and absorption coefficient change of GeSn. The change in the refractive index affects the optical mode in the cavity and thus the emission wavelength can be tuned. It is noteworthy that the similar strain engineering mechanism has been used for straining Si and Ge,^[^ ^22^, ^23^, ^24^, ^25^, ^26^, ^27^ ^]^ while the amount of the volume reduction in the pad areas is pre‐determined by the initial strain in the pad layer. The technique presented in this work can precisely control the amount of the tension in the pad on demand by harnessing controlled laser annealing, which will be further explained in a later section.

Figure 1b presents a cross‐sectional view of the schematic illustration (along the green dashed line in Figure 1a) before and after localized laser annealing. After the annealing, Sn atoms in the annealed area are segregated to the surface, as discussed in step II of Figure 1a, and Sn‐rich clusters are formed as shown in the right panel of Figure 1b.^[^ ^19^, ^20^, ^21^ ^]^ The insets to Figure 1b show atomic force microscopy (AFM) images of the pad before and after the annealing. The annealing condition will be described in detail later. While the unannealed surface is smooth, small dome‐shaped islands are observed on the annealed surface. The islands are Sn‐rich clusters produced by the segregated Sn,^[^ ^19^ ^]^ confirming that the Sn segregation occurs after the annealing. To further confirm whether the Sn segregation occurs by the annealing, we conducted Raman measurements (see Experimental Section for more details on Raman spectroscopy). Figure 1c shows Raman spectra measured at the pad before (blue) and after (red) the annealing. A spectrum of Ge bulk (black) showing a peak position at 300.6 cm^−1^ is also included as a reference. The peak positions of the spectra before and after the annealing are observed at ≈293.4 and ≈299.4 cm^−1^, respectively. From the peak shift of 6.0 cm^−1^ before and after annealing, a significant reduction of the Sn content by more than 8 at% is deduced using a Raman‐Sn content shift coefficient of 75 cm^−1^.^[^ ^28^ ^]^ This indicates that Sn atoms are removed from the GeSn lattice during annealing.

### 2.2 Theoretical and Experimental Verification of the Annealing‐Induced Strain

**Figure 2:**
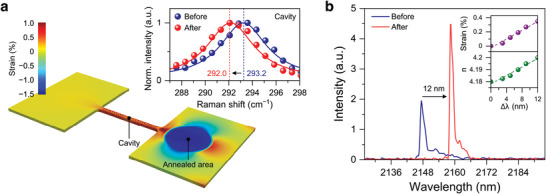
Theoretical and experimental verification of the annealing‐induced strain. a) Strain distribution of the annealed device calculated by FEM strain simulation. An annealed area is mimicked at the right‐side pad with an initial tensile strain of 1.3%. A tensile strain of ≈0.36% is shown at the center of the cavity. Inset: Raman spectra measured at the center of the cavity before (blue) and after (red) annealing. The peak positions of ≈293.2 and ≈292.0 cm^−1^ at the center of the cavity before and after the pad annealing, respectively, indicate the introduction of tensile strain in the laser gain medium. b) Experimental lasing spectra from the GeSn nanobeam laser before and after the pad annealing, showing a peak redshift of ≈12 nm. Inset: Calculated refractive index (bottom) and uniaxial tensile strain (top) as a function of emission wavelength shift.

To investigate how much the tensile strain is induced in the laser gain medium due to a pulling force in the tensioned pad, we theoretically calculated the strain of the annealed device via finite‐element method (FEM) strain simulation. We also experimentally measured tensile strain in the cavity by Raman spectroscopy and photoluminescence measurements and confirmed that the experimental strain is consistent with the theoretical strain. **Figure** **2**a shows a simulated strain distribution of the device containing the annealed pad area on the right side. To mimic the annealed area in the strain simulation, X‐ray diffraction (XRD) spectroscopy and FEM thermal simulation were additionally performed. Via XRD analysis, *T*_c_ was estimated to be ≈700 K (see Note S2, Supporting Information, for more details on the XRD analysis to determine *T*_c_). We then extracted an area exceeding 700 K through an FEM thermal simulation (see Note S3, Supporting Information, for more details on the thermal simulation to determine the annealed area). This allowed us to mimic the annealed region in the strain simulation. The initial tensile strain of the annealed area was set to be 1.3% estimated through strain calculation based on the change in lattice constant (see Note S4, Supporting Information, for more details on the calculation of tensile strain based on the change in lattice constant) and then the strain distribution was calculated with the fixed boundaries at the two ends of the pads. From the calculated strain distribution, a tensile strain of ≈0.36% is confirmed at the center of the cavity due to the pulling force in the annealed area. The inset to Figure 2a shows experimentally measured Raman spectra at the center of the cavity before (blue) and after (red) the annealing. The peaks are located at ≈293.2 and ≈292.0 cm^−1^ before and after annealing. The Raman peak is shifted by −1.2 cm^−1^ after the annealing, indicating that tensile strain is induced in the center of the cavity. Since there is currently no Raman‐uniaxial strain shift coefficient for GeSn with an Sn content of ≈10 at% in the literature, we investigated the amount of tensile strain by using photoluminescence.

Figure 2b shows lasing spectra from the GeSn nanobeam cavity before and after the annealing. All annealing experiments and lasing measurements were performed at 4 K. Prior to the annealing, the lasing spectrum was measured by pumping a 1550‐nm pulsed laser into the center of the nanobeam cavity with a pump power density of 98.1 kW cm^−2^ (see Experimental Section for more details on the laser characterization). The lasing characteristics showing clear lasing behavior of the GeSn nanobeam laser are provided in Figure S1, Supporting Information, (see Note S1, Supporting Information, for more details on the lasing characteristics of the GeSn laser). After obtaining the pre‐annealing spectrum, one of the suspended pads was annealed by the same 1550‐nm laser for ≈30 s with a pump power density of 28 MW cm^−2^, which is significantly larger than that used for obtaining lasing spectra (<100 kW cm^−2^). To achieve the Sn segregation with the laser annealing, the local temperature in the annealed area must rise above *T*_c_ of 700 K, which can be possible by using a very high peak power density of >14 MW cm^−2^ (see Experimental Section for more details on the laser annealing and Note S6, Supporting Information, for the detailed thermal simulations to confirm the device temperature distribution upon the laser annealing). After completing the annealing, the lasing spectrum was measured on the same device under the same pumping condition as the previous measurement. In Figure 2b, the post‐annealing spectrum shows a clear redshift of ≈12 nm compared with the pre‐annealing spectrum, confirming that tensile strain is induced in the gain medium. In addition, the emission intensity is increased after the annealing, which can be attributed to the improved directness in tensile‐strained GeSn.^[^ ^14^ ^]^ To deduce the amount of tensile strain in the gain medium, we compared the experimentally measured emission shift with the theoretically calculated emission shift for strained GeSn (see Note S5, Supporting Information, for more details on the calculation of tensile strain as a function of emission wavelength shift). The inset to Figure 2b presents a calculated <100> uniaxial tensile strain (top) and refractive index (bottom) as a function of emission wavelength shift. The refractive indices under varied peak positions are estimated by finite‐difference time‐domain (FDTD) simulations. We then calculated the strain using refractive index change by referring to a recent study.^[^ ^29^ ^]^ According to the theoretical calculations, the experimental peak shift of ≈12 nm corresponds to a uniaxial tensile strain of ≈0.35%. This result is in excellent agreement with our FEM strain simulation result discussed in Figure 2a, confirming the validity of our tuning approach.

### 2.3 Continuous Peak Shift with Dynamically Controlled Annealing

**Figure 3:**
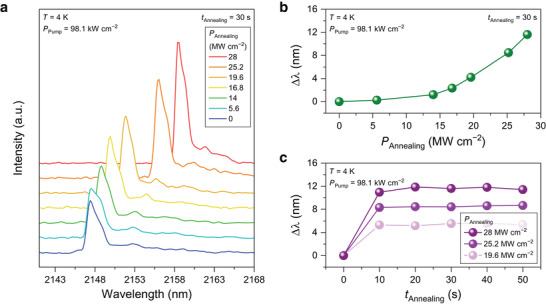
Continuous peak shift with dynamically controlled annealing. a) Lasing spectra measured with a power density of 98.1 kW cm^−2^ at 4 K under different optical annealing power *P*_annealing_. Annealing is conducted for 30 s for all annealing powers. The emission peak wavelength is shifted as *P*_annealing_ is increased, showing a shift of up to ≈12 nm with *P*_annealing_ of 28 MW cm^‐2^. b) Peak shift as a function of annealing power. c) Peak shift as a function of annealing time under annealing powers of 19.6, 25.2, and 28 MW cm^‐2^.

**Figure** **3**a shows lasing spectra from the same GeSn nanobeam laser under optical annealing with different annealing power densities. After obtaining the lasing spectrum prior to the annealing (blue), we used the same 1550‐nm laser to locally anneal in the pad area with different annealing laser power densities. The conditions for the annealing laser such as the duty cycle and pulse width were kept the same as those used for Figure 2b. The annealing was conducted for 30 s for all annealing power densities. The device prior to the annealing (blue) shows the spectrum with a peak position at 2148 nm. After the annealing with 14 MW cm^‐2^ (green), the lasing peak is redshifted by ≈1.5–2149.5 nm. The emission intensity is also increased possibly due to the improved directness enabled by the tensile strain induced in the gain medium.^[^ ^14^ ^]^ It is worth mentioning that the emission intensity can be controlled independently by adjusting the injection power density for lasing emission. After the annealing with higher pump power densities of 19.6 (orange) and 28 MW cm^‐2^ (red), the lasing peak is further shifted to longer wavelengths of 2152 and 2160 nm, respectively. The emission intensity keeps increasing as the peak is shifted to a longer wavelength, while the FWHM remains the same. The peak intensity is increased twice at the highest annealing power (red). To understand the origin of the continuous peak shift and the intensity increase upon the annealing at higher pump powers, we performed comprehensive thermal simulations. It is evident from the simulated thermal distributions (Figure S3, Supporting Information) that the annealed area exceeding *T*_c_ (700 K) is expanded at higher annealing power, which effectively increases the total volume reduction in the pad areas and the amount of induced strain in the gain medium (see Note S6, Supporting Information, for more details on the effect of the annealing pump powers).

Figure 3b shows the peak position shift as a function of the annealing power. The peak is shifted up to ≈12 nm continuously as the pump power is increased, showing the capability of precise emission tuning. A formula describing the relation between the annealing power and the shift in emission wavelength is also derived as explained in Note S8 (Supporting Information). Figure 3c shows peak shift as a function of the annealing time under different annealing powers. It is noteworthy that the lasing peaks shift to ≈5.5, 8.5, and 12 nm with annealing powers of 19.6, 25.2, and 28 MW cm^‐2^, respectively, within the first 10 s. The lasing peak positions for all annealing powers become saturated for any further annealing because the annealed area is not increased for prolonged annealing (see Note S7, Supporting Information, for more discussion).

### 2.4 Spectral Matching of Multiple Lasers via Precise Wavelength Tuning

**Figure 4:**
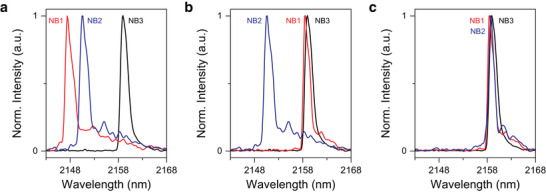
Spectral matching of multiple lasers via precise wavelength tuning. a) Normalized lasing spectra of three different GeSn nanobeam (NB) lasers before the spectral matching. b) Spectra after aligning NB1 to NB3. c) Spectra after aligning NB2 to NB1 and NB3. The variation of the peak positions between the three lasers after the spectral matching is only 0.39 nm.

As we confirmed the ability of our technique to achieve a continuous wavelength tuning, we conducted the last experiment to produce several laser sources emitting photons at the same wavelengths by precisely aligning the emission wavelengths of separate lasers. All measurements were performed at 4 K using the same pumping condition described in the Experimental Section. **Figure** **4**a presents the initial emission spectra of three nanobeam (NB) lasers showing lasing peak positions of ≈2148, ≈2151, and ≈2160 nm for NB1, NB2, and NB3, respectively. It is worth mentioning that although the three lasers were fabricated on a single GeSn‐on‐Si chip with the same fabrication process and design profile, they have different emission wavelengths due to the fabrication imperfection and the subtle variations of material properties at different locations on the chip. As shown in Figure 4b, the spectrum of NB1 is aligned to NB3 with a sub‐nanometer accuracy by using an annealing power of 28 MW cm^‐2^. The NB2 emission wavelength is then aligned to NB1 and NB3 by using an annealing power of 25.2 MW cm^‐2^. The variation of the peak positions between the three lasers is only 0.39 nm, demonstrating the great potential of our technique to achieve identical laser sources on a single chip. It should be noted that by using two separate external lasers, a pumping laser focused onto the cavity for getting the emission and an annealing laser focused onto the pads for shifting the emission peak, it is possible to monitor the peak shift in real‐time to further improve the spectral matching of separate devices.

## 3 Conclusion

In summary, we have demonstrated a scalable method to produce identical nanobeam lasers in a fully CMOS‐compatible GeSn‐on‐Si material system. This method involves dynamically controlled strain engineering to precisely tune the emission wavelengths of the GeSn lasers via localized annealing‐induced modification of GeSn crystal structure in a region far away from the laser gain medium. We have shown that emission wavelengths can be continuously tuned more than 10 nm, while the emission intensity increases twice and the FWHM remains the same. Using this method, we have demonstrated precise spectral matching of several laser devices whose initial emission wavelengths are considerably varied. We would like to note that this strain tuning is not reversible. Despite such a limitation, this permanent and precise spectral matching has various advantages. For example, once the spectral alignment is completed, the array of identical lasers can operate without consuming any additional energy for spectral tuning. This contrasts with other types of tuning mechanisms such as piezoelectric tuning, which requires a continuous supply of energy to maintain spectral matching.^[^ ^30^ ^]^ This spectral tuning scheme can become more convenient when it is applied to electrically pumped lasers,^[^ ^31^, ^32^ ^]^ since the laser annealing can be conducted on demand while monitoring electrically driven laser emission. Our simple and scalable technique to produce identical on‐chip lasers on a single chip opens up new avenues for realizing large‐scale PICs.

## 4 Experimental Section

### Raman Spectroscopy

A 100× objective lens was used to focus 532‐nm and Raman signals were collected through the same objective lens. All Raman measurements were performed at room temperature. To prevent heating effects during measurements, additional care was taken to maintain the pumping power low enough. The Raman‐Sn content shift coefficient of 75 cm^−1[^ ^28^ ^]^ was used to deduce the reduced Sn content in the annealed pad.

### Photoluminescence of the Laser Structures and Localized Optical Annealing

A 1550‐nm pump laser was used to characterize the laser devices. A pulse duration of 5 ns and a repetition rate of 1 MHz were set for the pumping laser, respectively. A 15× reflective objective was used to focus the pump laser into the center of the cavity and photoluminescence signals were collected through the same objective lens. The collected signals were guided to a Fourier transform infrared (FTIR) spectroscopy and detected by an extended InGaAs detector. The localized optical annealing was conducted by using the same 1550‐nm pump laser. To achieve a high peak power density that is crucial in increasing the local temperature above *T*_c_, a very low repetition rate of 250 kHz was used while the same pulse duration of 5 ns was employed.

## Conflict of Interest

The authors declare no conflict of interest.

## Supporting information

Supporting Information

## Data Availability

The data that support the findings of this study are available from the corresponding author upon reasonable request.

## References

[1] B. J. Shastri , A. N. Tait , T. Ferreira de Lima , W. H. P. Pernice , H. Bhaskaran , C. D. Wright , P. R. Prucnal , Nat. Photonics 2021, 15, 102.

[2] W. Bogaerts , D. Pérez , J. Capmany , D. A. B. Miller , J. Poon , D. Englund , F. Morichetti , A. Melloni , Nature 2020, 586, 207. 33028997 10.1038/s41586-020-2764-0

[3] P. Senellart , G. Solomon , A. White , Nat. Nanotechnol. 2017, 12, 1026. 29109549 10.1038/nnano.2017.218

[4] H.‐S. Zhong , H. Wang , Y.‐H. Deng , M.‐C. Chen , L.‐C. Peng , Y.‐H. Luo , J. Qin , D. Wu , X. Ding , Y. Hu , P. Hu , X.‐Y. Yang , W.‐J. Zhang , H. Li , Y. Li , X. Jiang , L. Gan , G. Yang , L. You , Z. Wang , L. Li , N.‐L. Liu , C.‐Y. Lu , J.‐W. Pan , Science 2020, 370, 1460. 33273064 10.1126/science.abe8770

[5] Y. Shen , N. C. Harris , S. Skirlo , M. Prabhu , T. Baehr‐Jones , M. Hochberg , X. Sun , S. Zhao , H. Larochelle , D. Englund , M. Soljačić , Nat. Photonics 2017, 11, 441.

[6] N. C. Harris , G. R. Steinbrecher , M. Prabhu , Y. Lahini , J. Mower , D. Bunandar , C. Chen , F. N. C. Wong , T. Baehr‐Jones , M. Hochberg , S. Lloyd , D. Englund , Nat. Photonics 2017, 11, 447.

[7] J. Q. Grim , A. S. Bracker , M. Zalalutdinov , S. G. Carter , A. C. Kozen , M. Kim , C. S. Kim , J. T. Mlack , M. Yakes , B. Lee , D. Gammon , Nat. Mater. 2019, 18, 963. 31285618 10.1038/s41563-019-0418-0

[8] E. Gil‐Santos , C. Baker , A. Lemaître , S. Ducci , C. Gomez , G. Leo , I. Favero , Nat. Commun. 2017, 8, 14267. 28117394 10.1038/ncomms14267 PMC5286200

[9] M. C. Y. Huang , Y. Zhou , C. J. Chang‐Hasnain , Nat. Photonics 2008, 2, 180.

[10] S. Wirths , R. Geiger , N. von den Driesch , G. Mussler , T. Stoica , S. Mantl , Z. Ikonic , M. Luysberg , S. Chiussi , J. M. Hartmann , H. Sigg , J. Faist , D. Buca , D. Grützmacher , Nat. Photonics 2015, 9, 88.

[11] D. Stange , S. Wirths , R. Geiger , C. Schulte‐Braucks , B. Marzban , N. von den Driesch , G. Mussler , T. Zabel , T. Stoica , J.‐M. Hartmann , S. Mantl , Z. Ikonic , D. Grützmacher , H. Sigg , J. Witzens , D. Buca , ACS Photonics 2016, 3, 1279. 10.1364/OE.24.001358 26832516

[12] J. Margetis , S. Al‐Kabi , W. Du , W. Dou , Y. Zhou , T. Pham , P. Grant , S. Ghetmiri , A. Mosleh , B. Li , J. Liu , G. Sun , R. Soref , J. Tolle , M. Mortazavi , S.‐Q. Yu , ACS Photonics 2018, 5, 827.

[13] J. Chrétien , N. Pauc , F. Armand Pilon , M. Bertrand , Q.‐M. Thai , L. Casiez , N. Bernier , H. Dansas , P. Gergaud , E. Delamadeleine , R. Khazaka , H. Sigg , J. Faist , A. Chelnokov , V. Reboud , J.‐M. Hartmann , V. Calvo , ACS Photonics 2019, 6, 2462.

[14] A. Elbaz , D. Buca , N. von den Driesch , K. Pantzas , G. Patriarche , N. Zerounian , E. Herth , X. Checoury , S. Sauvage , I. Sagnes , A. Foti , R. Ossikovski , J.‐M. Hartmann , F. Boeuf , Z. Ikonic , P. Boucaud , D. Grützmacher , M. El Kurdi , Nat. Photonics 2020, 14, 375.

[15] A. Elbaz , R. Arefin , E. Sakat , B. Wang , E. Herth , G. Patriarche , A. Foti , R. Ossikovski , S. Sauvage , X. Checoury , K. Pantzas , I. Sagnes , J. Chrétien , L. Casiez , M. Bertrand , V. Calvo , N. Pauc , A. Chelnokov , P. Boucaud , F. Boeuf , V. Reboud , J.‐M. Hartmann , M. El Kurdi , ACS Photonics 2020, 7, 2713.

[16] H.‐J. Joo , Y. Kim , D. Burt , Y. Jung , L. Zhang , M. Chen , S. J. Parluhutan , D.‐H. Kang , C. Lee , S. Assali , Z. Ikonic , O. Moutanabbir , Y.‐H. Cho , C. S. Tan , D. Nam , Appl. Phys. Lett. 2021, 119, 201101.

[17] Y. Kim , S. Assali , D. Burt , Y. Jung , H. Joo , M. Chen , Z. Ikonic , O. Moutanabbir , D. Nam , Adv. Opt. Mater. 2022, 10, 2101213.

[18] Y. Jung , D. Burt , L. Zhang , Y. Kim , H.‐J. Joo , M. Chen , S. Assali , O. Moutanabbir , C. Seng Tan , D. Nam , Photonics Res. 2022, 10, 1332.

[19] L. Wang , W. Wang , Q. Zhou , J. Appl. Phys. 2015, 118, 025701.

[20] P. Zaumseil , Y. Hou , M. A. Schubert , N. von den Driesch , D. Stange , D. Rainko , M. Virgilio , D. Buca , G. Capellini , APL Mater. 2018, 6, 076108.

[21] H. Cai , K. Qian , Y. An , G. Lin , S. Wu , H. Ding , W. Huang , S. Chen , J. Wang , C. Li , J. Alloys Compd. 2022, 904, 164068.

[22] M. J. Süess , R. Geiger , R. A. Minamisawa , G. Schiefler , J. Frigerio , D. Chrastina , G. Isella , R. Spolenak , J. Faist , H. Sigg , Nat. Photonics 2013, 7, 466.

[23] D. Nam , D. S. Sukhdeo , J.‐H. Kang , J. Petykiewicz , J. H. Lee , W. S. Jung , J. Vučković , M. L. Brongersma , K. C. Saraswat , Nano Lett. 2013, 13, 3118. 23758608 10.1021/nl401042n

[24] D. S. Sukhdeo , D. Nam , J.‐H. Kang , M. L. Brongersma , K. C. Saraswat , Photonics Res. 2014, 2, A8.

[25] J. Petykiewicz , D. Nam , D. S. Sukhdeo , S. Gupta , S. Buckley , A. Y. Piggott , J. Vučković , K. C. Saraswat , Nano Lett. 2016, 16, 2168. 26907359 10.1021/acs.nanolett.5b03976

[26] S. Bao , D. Kim , C. Onwukaeme , S. Gupta , K. Saraswat , K. H. Lee , Y. Kim , D. Min , Y. Jung , H. Qiu , H. Wang , E. A. Fitzgerald , C. S. Tan , D. Nam , Nat. Commun. 2017, 8, 1845. 29184064 10.1038/s41467-017-02026-w PMC5705600

[27] F. T. Armand Pilon , A. Lyasota , Y.‐M. Niquet , V. Reboud , V. Calvo , N. Pauc , J. Widiez , C. Bonzon , J. M. Hartmann , A. Chelnokov , J. Faist , H. Sigg , Nat. Commun. 2019, 10, 2724. 31222017 10.1038/s41467-019-10655-6 PMC6586857

[28] V. R. D'Costa , J. Tolle , R. Roucka , C. D. Poweleit , J. Kouvetakis , J. Menéndez , Solid State Commun. 2007, 144, 240.

[29] H. Tran , W. Du , S. A. Ghetmiri , A. Mosleh , G. Sun , R. A. Soref , J. Margetis , J. Tolle , B. Li , H. A. Naseem , S.‐Q. Yu , J. Appl. Phys. 2016, 119, 103106.

[30] O. Iff , D. Tedeschi , J. Martín‐Sánchez , M. Moczała‐Dusanowska , S. Tongay , K. Yumigeta , J. Taboada‐Gutiérrez , M. Savaresi , A. Rastelli , P. Alonso‐González , S. Höfling , R. Trotta , C. Schneider , Nano Lett. 2019, 19, 6931. 31486648 10.1021/acs.nanolett.9b02221

[31] Y. Zhou , Y. Miao , S. Ojo , H. Tran , G. Abernathy , J. M. Grant , S. Amoah , G. Salamo , W. Du , J. Liu , J. Margetis , J. Tolle , Y. Zhang , G. Sun , R. A. Soref , B. Li , S.‐Q. Yu , Optica 2020, 7, 924.

[32] B. Marzban , L. Seidel , T. Liu , K. Wu , V. Kiyek , M. H. Zoellner , Z. Ikonic , J. Schulze , D. Grützmacher , G. Capellini , M. Oehme , J. Witzens , D. Buca , ACS Photonics 2023, 10, 217.

