# Supplementary material for: High‐Precision Wavelength Tuning of GeSn Nanobeam Lasers via Dynamically Controlled Strain Engineering
Source: Adv Sci (Weinh). 2023 Apr 18;10(17):2207611. doi: 10.1002/advs.202207611 (PMC10265078; doi:10.1002/advs.202207611)
Supplement: Supplementary file 1 — Supporting Information [file ADVS-10-2207611-s001.pdf]

ADVANCED SCIENCE
Open Access

Supporting Information

for *Adv. Sci.*, DOI 10.1002/advs.202207611

High-Precision Wavelength Tuning of GeSn Nanobeam Lasers via Dynamically Controlled Strain Engineering

*Youngmin Kim, Hyo-Jun Joo, Melvina Chen, Bongkwon Son, Daniel Burt, Xuncheng Shi, Lin Zhang, Zoran Ikonic, Chuan Seng Tan and Donguk Nam**

Supporting Information

High-Precision Wavelength Tuning of GeSn Nanobeam Lasers via Dynamically Controlled Strain Engineering

*Youngmin Kim, Hyo-Jun Joo, Melvina Chen, Bongkwon Son, Daniel Burt, Xuncheng Shi, Lin Zhang, Zoran Ikonic, Chuan Seng Tan, and Donguk Nam**

Table of Content

Note 1. Device design, fabrication process and lasing characteristics

Note 2. XRD analysis to determine critical temperature

Note 3. FEM thermal simulation to determine annealed area

Note 4. Calculation of tensile strain based on the change in lattice constant

Note 5. Calculation of tensile strain as a function of emission wavelength shift

Note 6. Annealed area and tensile strain as a function of annealing power

Note 7. Temperature variation as a function of time

Note 8. Formula describing relation between annealing power and wavelength shift

References

Note 1. Device design, fabrication process and lasing characteristics

Design of a GeSn photonic crystal nanobeam cavity with two large pads: The width and length of the GeSn one-dimensional (1D) photonic crystal nanobeam cavity were designed to be 700 nm and 10μm, respectively. The photonic crystal was designed with the same approach as in our previous work.[1] The photonic crystal consists of periodic air holes with a lattice constant of 350 nm and three missing inner air holes at the center of the nanobeam. To reduce the scattering losses in the photonic crystal cavity by employing a gradual variation of the refractive index, the diameters of the first inner holes and the other holes are designed to be 112 and 217 nm, respectively. The two large pads connected to the cavity were designed with a width of 15 μm.

Fabrication of a GeSn nanobeam cavity with two large connected: Using GeSn-on-insulator (GeSnOI) substrate,[2,3] a GeSn photonic crystal nanobeam cavity was fabricated. Electron-beam lithography (EBL) was performed to pattern the nanobeam cavity and then Cl_2 dry etching with reactive ion etching (RIE) was conducted to transfer the pattern to the GeSn layer. To suspend the nanobeam and two large pads, the Al_2O_3 layer under the GeSn layer was selectively wet etched with 30 wt.% potassium hydroxide (KOH) at 80 °C. After the undercut of Al_2O_3 layer, HfO_2 thin film with a thickness of 90 nm was coated for the surface passivation through the atomic layer deposition (ALD) at 100 °C using tetrakis(ethylmethylamino)hafnium (TEMAH) and H_2O as the metalorganic and oxidation precursors, respectively.

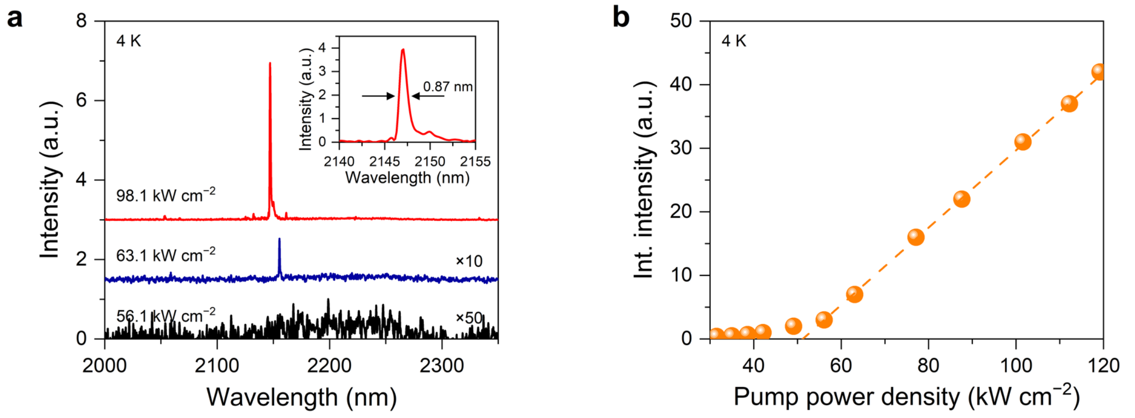

Figure S1. Lasing characteristics from unannealed GeSn nanobeam laser at 4 K. a) Emission spectra as a function of pump power. Inset: magnified view of the spectrum with a pump power density of 98.1 kW cm^{-2}, showing a narrow FWHM of 0.87 nm. b) L-L curves taken at 4 K (threshold, 51.2 kW cm^{-2}).

Lasing characteristics: To investigate lasing characteristics from the fabricated devices, we conducted photoluminescence measurements on an unannealed GeSn nanobeam laser. **Figure S1a** shows the emission spectra of the GeSn laser pumped with the power densities of 56.1, 63.1 and 98.1 kW cm^{-2}. At a low pump power density of 56.1 kW cm^{-2}, only broad spontaneous emission is observed. As the pump power density increases to 63.1 kW cm^{-2}, a single sharp lasing peak appears at the wavelength of 2150 nm. At a higher pump power density of 98.1 kW cm^{-2}, a clear lasing peak is shown with a very narrow full width at half-maximum (FWHM) of 0.87 nm, as shown in the inset. Further evidence of lasing can be seen in Figure S1b, where the light-in-light-out (L-L) curve of the GeSn nanobeam laser exhibits clear threshold behavior as the pump power density increases. The threshold power density is determined to be 51.2 kW cm^{-2}.

Note 2. XRD analysis to determine critical temperature

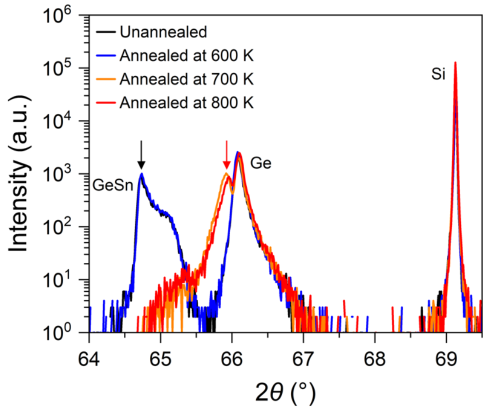

Figure S2. Specular 2θ-ω scans of (004) diffraction for unannealed and annealed GeSnOI samples. Annealing was conducted at temperatures of 600, 700 and 800 K by RTA. The black and red arrows mark the positions of the GeSn diffraction peaks for all samples. The unannealed sample and the sample annealed at 600 K show the same GeSn diffraction peak at ~64.7°, indicating that the Sn segregation is not invoked at 600 K. The samples annealed at 700 and 800 K show the same GeSn diffraction peaks at ~65.9 °. The GeSn diffraction peaks of 700 and 800 K samples are closer to the Ge diffraction peak, indicating that Sn segregation is invoked beyond 700 K. Via XRD analysis, T_c is estimated to be 700 K.

To determine the critical temperature (T_c) that induces the Sn segregation in our GeSn with an Sn content of ~10 at%, X-ray diffraction (XRD) spectroscopy measurements were performed for unannealed and annealed GeSnOI samples. Rapid thermal annealing (RTA) was performed on GeSnOI, the same substrate used for the tuning experiments, at temperatures of 600, 700 and 800 K. **Figure S2** shows specular 2θ-ω scans of (004) diffraction of unannealed and annealed samples. The GeSn diffraction peak positions for all samples are marked by black and red arrows. The unannealed sample and the sample annealed at 600 K show the same GeSn diffraction peaks at ~64.7° corresponding to the Sn content of ~10 at%, indicating that Sn is not segregated by the temperature of 600 K. For the samples annealed at 700 and 800 K, the GeSn diffraction peaks are observed at the same position of ~65.9° corresponding to the Sn content of ~1 at%. The GeSn peaks are very closer to the Ge diffraction peak, indicating a significant reduction of the Sn content after annealing at 700 and 800 K. According to these results, we estimated the T_c as 700 K.

Note 3. FEM thermal simulation to determine annealed area

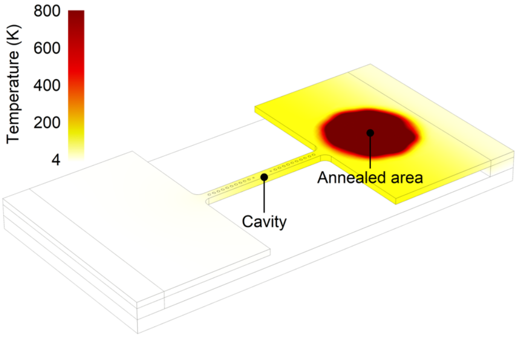

Figure S3. Thermal distribution of the device during the pad annealing calculated by FEM thermal simulation. A pulsed heat source mimicking the pumping condition used in the annealing experiments was applied to one of the suspended pads. The area exceeding T_c (700 K) is calculated to be ~28 μm^2.

To determine an annealed area by localized laser annealing in our device, we performed a thermal simulation using finite-element method (FEM). **Figure S3** shows a thermal distribution simulated with a heat source mimicking the pumping condition used in the annealing experiments. A pulsed heat source with a pump power density of 28 MW cm^{-2} was applied at one of the suspended pads. The repetition rate and pulse duration were set to 250 kHz and 5 ns, respectively. The base temperature was set to 4 K. From the calculated thermal distribution, an area exceeding T_c (700 K) can be seen at the heated pad. The area is calculated as ~28.2 μm^2 and we set this area as an annealed area in the strain simulation. It is noteworthy that the temperature rise occurs only in a very localized area, preventing temperature-induced changes in other parts of the devices.

Note 4. Calculation of tensile strain based on the change in lattice constant

Lattice constant of GeSn with different Sn composition

The lattice constant of GeSn (a_{GeSn}) as a function of Sn composition can be written as:[4]

$$a_{GeSn} = a_{Sn}x + a_{Ge}(1-x) + 0.00882(1-x), \quad (1)$$

where a_{Sn} and a_{Ge} are the lattice constants of Sn and Ge, and the values are 6.4892 and 5.6579 Å,[5] respectively, x is the Sn composition of GeSn. Using equation (1), the lattice constants of $Ge_{0.90}Sn_{0.10}$ and $Ge_{0.99}Sn_{0.01}$ discussed in Supporting Information Note 2 are calculated to be 5.7490 and 5.6749 Å.

Strain in the Sn segregated area based on the change in lattice constant

Strain is defined as a change in dimension divided by the original dimension. In our system, the lattice of $Ge_{0.99}Sn_{0.01}$ should be formed in the annealed area due to Sn segregation, but in reality the atoms are rearranged in the lattice of $Ge_{0.90}Sn_{0.10}$. Consequently, the strain based on the change in lattice constant can be written as:

$$\varepsilon_{\parallel} = \frac{a_{Ge_{0.90}Sn_{0.10}} - a_{Ge_{0.99}Sn_{0.01}}}{a_{Ge_{0.99}Sn_{0.01}}}, \quad (2)$$

By employing lattice constants of $Ge_{0.90}Sn_{0.10}$ and $Ge_{0.99}Sn_{0.01}$ calculated above, we obtain in-plane tensile strain of ~1.3%.

Note 5. Calculation of tensile strain as a function of emission wavelength shift

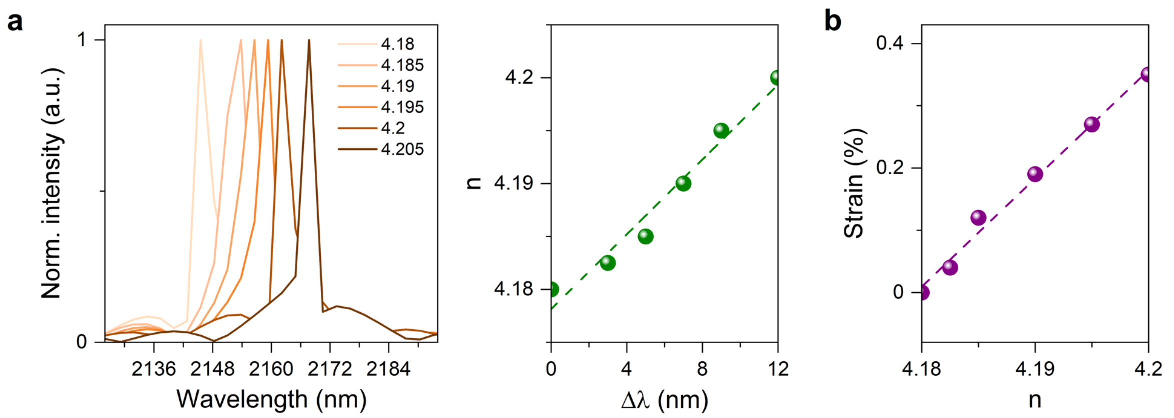

Figure S4. Calculation of tensile strain as a function of emission wavelength. a) Simulated spectra (left panel) of our structure with varied refractive indices using FDTD simulation. The peak shift corresponding to each refractive index is shown in the right panel b) Theoretically calculated <100> uniaxial tensile strain as a function of refractive index.

To investigate how much the tensile strain is induced in the laser gain medium, we conducted finite-difference time-domain (FDTD) optical simulations and theoretical calculation. **Figure S4**a shows simulated spectra (left panel) of our 1D photonic crystal nanobeam cavity structure with varied refractive indices using FDTD simulation. The peak shift corresponding to each refractive index is also shown in the right panel. The simulated spectra show a single mode for all refractive indices, indicating no mode hopping in our structure. From the peak shift with varied refractive indices, we confirmed that the peak shifts by ~12 nm for 0.02 index increase. We then calculated the strain using refractive index change by referring to a recent study.[6] Figure S4b shows a theoretically calculated <100> uniaxial tensile strain as a function of refractive index. From the calculation, we confirmed that the change in refractive index of 0.02 induces tensile strain of ~0.35%. Consequently, the experimental peak shift of ~12 nm corresponds to a uniaxial tensile strain of ~0.35%. This result is in excellent agreement with our FEM strain simulation result discussed in Figure 2a, confirming the validity of our tuning approach.

Note 6. Annealed area and tensile strain as a function of annealing power

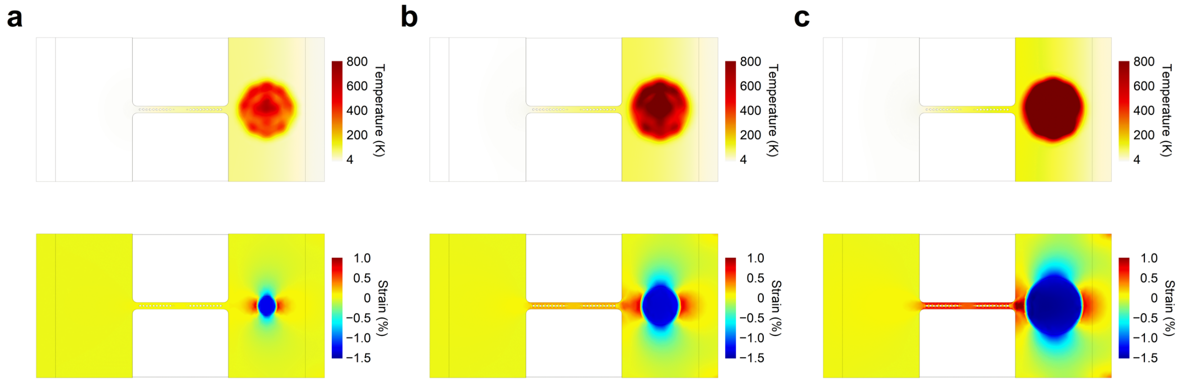

Figure S5. Annealed area and tensile strain as a function of annealing power. Thermal (top) and strain (bottom) distributions for the annealing power densities of a) 14 b) 19.6 and c) 28 MW cm^{-2} calculated using FEM. The annealed area exceeding T_c of 700 K is expanded as the annealing power increases. The annealed areas were estimated to ~1.5, ~7.1 and ~28.3 μm^2 by the thermal distributions. Tensile strains of ~0.02, ~0.09 and ~0.35% are calculated at the center of the cavity by strain simulations using the estimated annealed area.

To investigate how the higher annealing power achieves a longer wavelength shift, we performed FEM thermal and strain simulations. **Figure S5** presents calculated thermal (top) and strain (bottom) distributions with the annealing power densities of 14 (Fig. S5a), 19.6 (Fig. S5b) and 28 MW cm^{-2} (Fig. S5c). It is evident from the thermal distributions that the annealed area exceeding T_c (700 K) is expanded as the annealing power increases. By calculating the area exceeding T_c, we could estimate the annealed areas of ~1.5, ~7.1 and ~28.3 μm^2 for the annealing power densities of 14, 19.6 and 28 MW cm^{-2}, respectively. We employed these areas as annealed areas in the strain simulation and set the initial tensile strain of 1.3% by referring to the calculation discussed in Supporting Information Note 4. Tensile strains of ~0.02, ~0.09 and ~0.35%, corresponding to theoretical emission wavelength shifts of ~1.5, ~8, 12 nm (Figure 2b inset), are calculated at the center of the cavity via strain simulations. These results are in excellent agreement with our experimentally measured emission peak shifts under annealing power densities of 14, 19.6 and 28 28 MW cm^{-2} (Figure 3b), explaining how we achieved the longer wavelength shifts with the higher annealing powers.

Note 7. Temperature variation of annealing region as a function of time

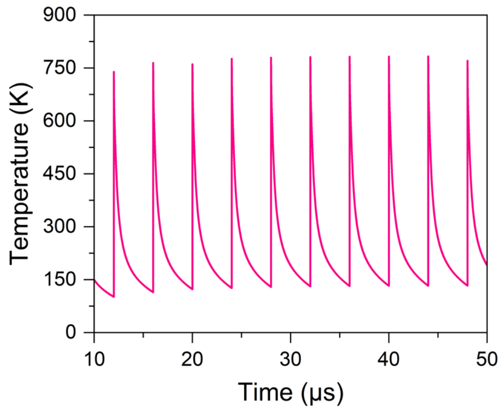

Figure S6. Temperature variation of annealing region as a function of time. Pulsed pumping with a pulse width of 5 ns and repetition rate of 250 kHz is applied. During the pulse duration of 5 ns, the temperature reaches a maximum of ~750 K. Until the next pulse, the temperature reaches a minimum of ~130 K due to the heat dissipation. It is evident that there is no heat accumulation over time.

To understand the fixed peak shift of ~12 nm for varied annealing times (Figure 3c), we analyzed the temperature of the annealing region over time in Fig. S3a. **Figure S6** shows the simulated temperature of the annealing region as a function of time. Due to the pulsed pumping with a pulse width of 5 ns and repetition rate of 250 kHz, the temperature increases and decreases periodically. During pulse duration of 5 ns, the pumping is induced and the temperature reaches a maximum of ~750 K over the T_c (700 K). Until the next pulse, the heating dissipates and the temperature reaches a minimum of ~130 K, which is much lower than T_c. This rising and falling of the temperature is repeated every pumping period without any heating accumulation over time. This indicates that the way our devices are annealed is not a thermal accumulation that takes a long time to complete the annealing process. It can further be deduced that the annealing process can be completed for the short pulse peak duration of 5 ns. According to the analysis, the annealing times performed in our experiments (Figure 3c) do not show any time-dependent effect, possibly because the annealing process may be completed before 10 s.

Note 8. Formula describing relation between annealing power and wavelength shift

We derived an empirical formula that correlates annealing power with the shift in emission wavelength. The formula is modeled as an exponential function because the relation between annealing power and strain shows an exponential correlation in the thermal and strain simulations (see Supporting Information Note 6 for more details on annealing power-dependent strain in our device). On the other hand, the other relations such as strain vs. refractive index and refractive index vs. wavelength shift show a linear correlation (see Supporting Information Note 5 for more details on strain-dependent refractive index and refractive index-dependent wavelength shift). Therefore, the emission wavelength changes exponentially as a function of the annealing power. We fitted an exponential equation to the data points acquired through experiments for annealing power-dependent wavelength shift in Figure 3b. As a result, the formula describing the relation between annealing power and wavelength shift can be written as follows:

$$\Delta\lambda = 0.26293\left(e^{\frac{P_{Annealing}}{7.29411}} - 1\right)$$

References

[1] H.-J. Joo, Y. Kim, D. Burt, Y. Jung, L. Zhang, M. Chen, S. J. Parluhutan, D.-H. Kang, C. Lee, S. Assali, Z. Ikonic, O. Moutanabbir, Y.-H. Cho, C. S. Tan, D. Nam, *Appl. Phys. Lett.* **2021**, *119*, 201101.
[2] B. Wang, E. Sakat, E. Herth, M. Gromovyi, A. Bjelajac, J. Chaste, G. Patriarche, P. Boucaud, F. Boeuf, N. Pauc, V. Calvo, J. Chrétien, M. Frauenrath, A. Chelnokov, V. Reboud, J.-M. Hartmann, M. El Kurdi, *Light Sci. Appl.* **2021**, *10*, 232.
[3] D. Burt, H.-J. Joo, Y. Jung, M. Chen, Y.-C. Huang, D. Nam, *Opt. Express* **2021**, *29*, 28959.
[4] N. Bhargava, M. Coppinger, J. Prakash Gupta, L. Wielunski, J. Kolodzey, *Appl. Phys. Lett.* **2013**, *103*, 041908.
[5] M. Oehme, J. Werner, M. Gollhofer, M. Schmid, M. Kaschel, E. Kasper, J. Schulze, *IEEE Photonics Technol. Lett.* **2011**, *23*, 1751.
[6] H. Tran, W. Du, S. A. Ghetmiri, A. Mosleh, G. Sun, R. A. Soref, J. Margetis, J. Tolle, B. Li, H. A. Naseem, S.-Q. Yu, *J. Appl. Phys.* **2016**, *119*, 103106.
